# Mitochondria in Acetaminophen-Induced Liver Injury and Recovery: A Concise Review

**DOI:** 10.3390/livers3020014

**Published:** 2023-04-10

**Authors:** Anup Ramachandran, Hartmut Jaeschke

**Affiliations:** Department of Pharmacology, Toxicology, and Therapeutic, University of Kansas Medical Center, Kansas City, KS 66160, USA;

**Keywords:** acetaminophen, mitochondria, paracetamol, iron, morphology, spheroid, biogenesis

## Abstract

Mitochondria are critical organelles responsible for the maintenance of cellular energy homeostasis. Thus, their dysfunction can have severe consequences in cells responsible for energy-intensive metabolic function, such as hepatocytes. Extensive research over the last decades have identified compromised mitochondrial function as a central feature in the pathophysiology of liver injury induced by an acetaminophen (APAP) overdose, the most common cause of acute liver failure in the United States. While hepatocyte mitochondrial oxidative and nitrosative stress coupled with induction of the mitochondrial permeability transition are well recognized after an APAP overdose, recent studies have revealed additional details about the organelle’s role in APAP pathophysiology. This concise review highlights these new advances, which establish the central role of the mitochondria in APAP pathophysiology, and places them in the context of earlier information in the literature. Adaptive alterations in mitochondrial morphology as well as the role of cellular iron in mitochondrial dysfunction and the organelle’s importance in liver recovery after APAP-induced injury will be discussed.

## 1. Introduction

Mitochondria are unique organelles that evolved from the integration of an endosym-biotic alphaproteobacterium into a host eukaryotic cell of the Archaea group [1]. This symbiotic relationship was refined over millions of years of evolution, and these organelles are now critical to eukaryotic cell function. Mitochondria are essential to several cellular functions and have been extensively studied over the years, with emphasis on their role in cellular energy maintenance through ATP generation. In addition, mitochondria are critical hubs of cellular metabolism, being involved in glucose metabolism to produce acetyl CoA, as well as fatty acid oxidation and redox homeostasis [2], among others. These critical functions are possible due to the unique structural features of mitochondria, which are double membrane structures with inner membrane folds called cristae that increase their surface area and accommodate the protein complexes of the electron transport chain (ETC) [3]. This dual membrane structure and localization of the ETC components on the inner mitochondrial membrane are important for the establishment of a proton motive force during electron transport for ATP synthesis [3]. The outer mitochondrial membrane, on the other hand, functions as a diffusion barrier for small molecules and helps create rate-dependent concentration gradients for metabolic function [4]. Additionally, outer membrane proteins also play critical roles in mitochondrial fusion and fission as well as mitophagy [5]. Another unique feature of mitochondria is the presence of mitochondrial DNA in the matrix within the organelle. Several copies of this double-stranded circular genome are present within the matrix, encoding 13 proteins of the ETC along with mitochondria-specific ribosomal RNA and tRNA [6].

In recent decades, the role of mitochondria in cellular signaling and its control of the cellular response to various pathophysiological conditions have been intensely investigated. While this was initially focused on cell death pathways such as apoptosis, it is now evident that mitochondria are essential organelles in almost all facets of cellular homeostasis and signaling, especially in specialized cells with energy-intensive functions and abundant mitochondria such as hepatocytes. This is especially important from the standpoint of drug metabolism and its consequences since it is recognized that critical enzymes in the process such as cytochrome P450 2E1 can localize to mitochondria [7,8]. While CYP450 enzymes are essential mediators of drug metabolism and xenobiotic scavenging under homeostasis, the inadvertent formation of reactive metabolites during metabolism plays a critical role in drug-induced pathophysiology. One of the most clinically relevant examples is hepatotoxicity induced by an acetaminophen (APAP) overdose, which is the most common cause of acute liver failure (ALF) in the United States and many western countries [9,10], where hepatocyte mitochondria play a central role in pathophysiology [11]. This concise review examines recent evidence that has uncovered the nuanced role played by this critical organelle in regulating cellular decision-making in response to an APAP overdose.

## 2. Acetaminophen Metabolism and Early Mitochondrial Insults

Generally, therapeutic doses of APAP are not toxic due to rapid metabolism in hepatocytes to glucuronide or sulphate metabolites by UDP-glucuronosyl transferases or sulfotransferases, respectively [12]. While a minor percentage of a therapeutic APAP dose is also metabolized by cytochrome P450 enzymes such as Cyp2E1, Cyp1A2, and Cyp3A4 to form a reactive metabolite, N-acetyl-p-benzoquinone imine (NAPQI) [12], this is efficiently scavenged by hepatic glutathione and hence does not induce cellular damage. A minor formation of protein adducts by therapeutic doses is effectively removed by autophagy [13,14]. However, some conditions, such as fatty liver disease, could predispose patients to APAP hepatotoxicity [15]. In fact, patients with pre-existing conditions such as severe acute viral hepatitis or those on antitubercular drugs have been shown to exhibit features of liver injury even after therapeutic doses of APAP [16]. A recent study from France indicated that patients with excess drinking and/or fasting exhibited liver injury on therapeutic doses of APAP (defined as <6 g/d in the study) [17]. The increased susceptibility to injury in alcoholics is probably due to the compromised mitochondrial structure and function evident in this population [18–20] while fasting interferes with glutathione resynthesis and thus facilitates injury. In addition to these vulnerable populations, a randomized controlled trial also indicated transient elevations in aminotransferase levels in healthy adults receiving a therapeutic dose of 4 g APAP daily [21]. However, there was never any severe liver injury or ALF in these patients [21]. The possible mechanisms involved in this benign ALT elevation are not very clear, and this is an area that deserves to be investigated in detail. Biomarkers are being identified that can distinguish between hazardous and benign ALT elevations, and some of them are derived from mitochondria, e.g., argininosuccinate synthase 1 [22].

While the rate of glucuronidation can be significantly upregulated in response to an APAP overdose [23], this seems to be insufficient to prevent shunting of APAP towards cytochrome P450-mediated reactive metabolite formation after an overdose, probably due to limitations in the availability of UDP-glucuronic acid. The enhanced cytochrome P450-mediated formation of NAPQI is central to APAP pathophysiology in the liver [24], and the subsequent depletion of hepatic glutathione stores initiates a complex signaling cascade where the mitochondria take center stage. Even though reactive metabolites such as NAPQI could presumably react with a multitude of cellular cysteine- or lysine-containing proteins after glutathione depletion [12], the formation of mitochondrial protein adducts is critical for APAP-induced hepatotoxicity [25,26]. While it was generally believed that the formation of mitochondrial protein adducts immediately initiated hepatocytes on a slippery slope of cell death signaling culminating in hepatocyte necrosis, recent evidence indicates that the mitochondrial response is much more nuanced, with the initial response being attempts at adaptation prior to commitment towards cell death.

## 3. Adaptive Mitochondrial Response and Changes in Mitochondrial Morphology

Excessive formation of NAPQI targets hepatocyte mitochondrial proteins for adduct formation, which severely compromises protein function and subsequently induces mitochondrial oxidative stress [27]. However, initial superoxide formation due to adducts on complex III is directed away from the mitochondrial matrix and inner membrane towards the intermembrane space and cytosol, preserving mitochondrial respiratory chain function [28]. Subsequent JNK activation and mitochondrial translocation in hepatocytes amplifies mitochondrial oxidative stress, as will be detailed in the next section, but the initial decrease in mitochondrial membrane potential only seems to activate a mitochondrial adaptation by change in morphology. Changes in mitochondrial morphology and dynamics have been well recognized as being important during various phases of cellular metabolism, and their role in APAP-induced hepatocyte necrosis is also recognized [29]. While most changes in mitochondrial morphology contributing to mitochondrial dynamics (mitochondrial fission and fusion) are controlled by proteins such as mitofusins and Drp1 [29], it is recognized that changes in mitochondrial morphology can also occur independently of these canonical pathways [30–32]. These studies have identified donut-like or spheroid mitochondria, which seem to be produced in response to changes in mitochondrial membrane potential or mitochondrial oxidative stress [30,33]. Our work on early mitochondrial changes in hepatocytes after APAP exposure also revealed the formation of donut-like mitochondria accompanied by loss of mitochondrial membrane potential without a significant effect on mitochondrial respiratory rates [34]. Interestingly, these changes were reversible on removal of APAP [34], such as those seen after hypoxia-induced reoxygenation in cardiomyocytes [33]. Similar transitions from tubular to donut-shaped mitochondria were also reversible when lung epithelial cells were treated with inhibitors of mitochondrial respiration with the generation of reactive oxygen species [35]. While mitochondrial generation of reactive oxygen species has been implicated for decades in APAP-induced hepatocyte necrosis [36], it is now revealed that early superoxide generation, which probably accompanies the loss of mitochondrial membrane and formation of donut-like mitochondria, occurs without proton leak from the mitochondrial respiratory chain or alteration in mitochondrial electron transport.

A detailed study using confocal microscopy and 3D electron microscopic tomography of this mitochondrial morphology change induced by loss of mitochondrial membrane potential revealed that these donut-like mitochondria have central indentations forming discoid forms while lacking holes in the center [30]. The mechanisms involved in the formation of such discoid mitochondria have not been characterized, but it has been suggested to occur through physical membrane mechanisms to attain a final structure with the lowest free energy [27], though membrane phospholipids could also play a role [37]. Computational analysis also indicates that formation of the discoid shape is facilitated by the release of osmotic potential energy through a decrease in total Gibbs free energy, with the bending energy being the barrier for donut formation [38]. Another factor that has been implicated in mitochondrial discoid formation is cellular calcium dynamics, with the transition to the donut shape being mediated by the mitochondrial Miro1 protein in a calcium-dependent manner [32]. Interestingly, increases in cytosolic calcium have been noted in cultured hepatocytes after treatment with APAP [39] within time frames where donut-shaped mitochondria were also detected [34]. This change in intracellular calcium has also been implicated in APAP-induced hepatotoxicity [40], suggesting that the effect of intracellular calcium on this adaptive mitochondrial morphology response could have consequences for downstream cellular signaling, though that is an area for further investigation. Additionally, adaptive changes in mitochondrial bioenergetics such as those induced by enhanced respiratory chain flux in mice deficient in pyruvate dehydrogenase kinase 4 (PDK4) can render them highly efficient in handling APAP-induced oxidant stress, probably through modulation of UCP2 levels [41].

Given the central role of hepatocyte mitochondria in the injury process, additional adaptive mechanisms that mitigate this effect have been recognized. Most important is the process of autophagy or, more specifically, mitophagy, which can remove damaged mitochondria and thereby limit the progression of the cell death mechanisms during APAP hepatotoxicity [42]. Damaged mitochondria are identified through the PINK1/Parkin pathway [43–45] but may also involve Parkin-independent mechanisms [46,47]. It was shown that autophagy is activated after a single APAP overdose and that removal of damaged mitochondria [48] and protein adducts [13] attenuated APAP-induced liver injury. However, autophagy appears to be most effective in cells located at the outer area of necrosis, where the severity of the insult is more limited and adaptive mechanisms have a chance to successfully intervene [49]. Consistent with this hypothesis is the observation that the removal of cytosolic and mitochondrial protein adducts by autophagy is most effective in preventing liver injury at therapeutic or moderately supratherapeutic doses of APAP [14]. Because even these low doses of APAP can cause protein adduct formation in humans [50], the consistent removal of these adducts by autophagy makes it possible that therapeutic doses of APAP can be used chronically for years without adverse effects. In animal studies, it was shown that therapeutic doses of APAP mainly cause soluble protein adduct formation, while repeated supratherapeutic doses or a severe overdose also cause mitochondrial adduct formation [14]. However, any inhibition of autophagy can rapidly trigger liver injury after multiple therapeutic or supratherapeutic doses, demonstrating the vital importance of the autophagy/mitophagy processes for cell survival [14].

## 4. Activation of the MAP Kinase Cascade and Amplification of Mitochondrial Injury Cause Hepatocyte Necrosis

Persistent activation of JNK in the cytosol and the translocation of phosphorylated JNK to the mitochondria overcomes the adaptive mitochondrial mechanisms to ultimately amplify mitochondrial oxidative stress and compromise mitochondrial respiration. Phosphorylated JNK binds to the mitochondrial outer membrane protein Sab [51] and inhibits the electron transport chain (ETC) through a Src-dependent process [52]. Among the JNK isoforms, experiments with anti-sense oligonucleotides targeting them individually indicate that JNK2 is probably more important in APAP hepatotoxicity, though JNK1 can take over in its absence, indicating both isoforms are involved. JNK activation is also important to APAP pathophysiology in primary human hepatocytes [53], though it seems to be inconsequential to cell death pathways after APAP exposure in the human hepatoma HepaRG cell line [53,54], which exhibit all other signaling characteristics after APAP [53]. The demonstration of JNK phosphorylation in human liver tissue, however, is complicated by the timing of sample availability since liver biopsies are typically contraindicated in APAP overdose patients in the clinic due to the risk of bleeding. The few studies examining liver biopsies from APAP overdose patients only collect them after coagulation parameters have stabilized [55,56], but these may not be very useful since JNK activation after APAP is transient [57] and unlikely to be detected at these later time points.

JNK interaction with mitochondrial Sab and the inhibition of the ETC then results in elevated mitochondrial free radical generation, which is now also derived from complex I [58], unlike the initial superoxide generation from complex III [28]. Mitochondrial respiratory complex II (succinate dehydrogenase) has been identified as a sensitive target for NAPQI-mediated inhibition of activity [59], which would have significant effects on energy homeostasis through modulation of the TCA cycle since succinate dehydrogenase participates in both the TCA cycle and ETC [60]. However, this is likely a later event after JNK translocation, based on studies in cultured cells and in vivo experiments [27]. This would be especially important considering the recent report suggesting that mitochondrial complex I is dispensable for the homeostasis of the adult mouse liver, which compensates through alternate electron donors to fuel the mitochondrial ETC [61]. Thus, inhibition of a predominant alternative electron donor such as complex II by APAP could have dramatic effects on hepatic mitochondrial function. This is highlighted by the demonstration that use of methylene blue to accept electrons from NAPQI-modified complex II and transfer them to cytochrome c, bypassing this inhibition, prevented hepatocyte necrosis [62]. However, complex II inhibition was not detected by in vitro respiratory measurements in human liver biopsies exposed to APAP [63], and the clinical use of methylene blue would require tight control of dosage due to the severe risk of methemoglobinemia as a side effect of this drug [64].

To prevent cellular injury in the event of enhanced free radical generation from the organelle, mitochondria have active antioxidant systems such as the manganese superoxide dismutase (MnSOD), which typically scavenge excess free radicals. These systems are critical to APAP pathophysiology since partial deficiency of MnSOD exacerbates APAP-induced liver injury [65,66]. Mitochondrial dysfunction is amplified when these anti-oxidant systems are compromised by the formation of the potent oxidant peroxynitrite [67] after the reaction of superoxide with nitric oxide (NO) within the cellular compartment [68]. Interestingly, the initial superoxide generation from mitochondria into the cytosol is not accompanied by peroxynitrite formation [28], which is only evident after JNK translocation to the mitochondria with active inhibition of the mitochondrial respiratory chain [69,70]. This further suggests that the source of NO for the formation of peroxynitrite in the context of APAP pathophysiology is likely within the mitochondria, since nitrotyrosine adducts are only detected inside mitochondria [68] and peroxynitrite formation is not evident when superoxide is shunted towards the cytosol [28]. The most likely contributor of NO within mitochondria seems to be neuronal nitric oxide synthase (nNOS), since its deficiency prevented APAP-induced liver injury without affecting metabolism [67] and it has been suggested to be localized to mitochondria [71,72], though this is controversial [73]. Irrespective of the source of NO, mitochondrial peroxynitrite formation is central to APAP pathophysiology, as demonstrated by the robust protection provided by interventions targeting its formation, such as the use of the SOD mimetic Mito-TEMPO [74,75] or its scavenging with GSH [76]. An additional role for cellular iron in peroxynitrite toxicity in mitochondria in the context of APAP pathophysiology has now been revealed by recent research [77]. While the role of cellular iron in APAP pathophysiology has been controversial [77], its nuanced contribution has been indicated in more recent studies examining lysosomal instability after APAP overdose [78]. Release of lysosomal iron into the cytosol [79] and its uptake into mitochondria [80] were noted, and the importance of these phenomena to the pathophysiology was evidenced by the protection against cell necrosis conferred by chelation of lysosomal iron or blocking its mitochondrial uptake [79–81]. We recently showed that treatment with deferoxamine and minocycline did not influence activation and translocation of JNK but prevented the formation of nitrotyrosine protein adducts from peroxynitrite and subsequent steps such as induction of the mitochondrial permeability transition [77]. Since iron can facilitate the formation of nitrotyrosine from peroxynitrite in a milieu of GSH depletion [82], this indicates that mitochondrial iron accumulation from the lysosome facilitates mitochondrial amplification of injury.

An important consequence of mitochondrial peroxynitrite formation is the damage to mitochondrial DNA, seen within 3 h after a dose of 300 mg/kg APAP [68]. This causes substantial depletion of mtDNA within the liver [68] and release of mtDNA fragments into the circulation in mice and humans [83]. These DNA fragments could function as damage-associated molecular patterns (DAMPs) to initiate the innate immune response necessary for liver recovery and regeneration [83]. The loss of mtDNA would have catastrophic effects on the maintenance of mitochondrial homeostasis in damaged hepatocytes, not only impacting ATP synthesis but also decreasing fatty acid oxidation [6]. This has been evident after an APAP overdose [84] and was initially identified in serum metabolomic studies that revealed marked elevations in serum acylcarnitine early after an APAP overdose in mice, which were prevented in Cyp2E1-deficient mice [85]. Interestingly, these changes were not evident at a lower (200 mg/kg) overdose of APAP, which induces all other signaling pathways and liver necrosis [86], but only evident at the higher 400 mg/kg overdose of APAP in 129/Sv mice [85]. This indicates that severe mitochondrial dysfunction is probably required for these changes in lipid metabolism to occur after APAP. However, there seems to be strain-dependent differences in this effect since B6C3F1 mice showed early elevations in circulating acylcarnitines even at the 200 mg/kg dose [87], similar to C57BL6/J mice [88]. The role of this inhibition of fatty acid oxidation in APAP pathophysiology was also highlighted by a recent study, which showed that hepatocyte-specific activation of the G protein-coupled receptor Mas, involved in the renin-angiotensin system, enhanced lipophagy and fatty acid oxidation to protect against APAP-induced hepatotoxicity in mice [89]. The APAP-induced inhibition of fatty acid oxidation also seems to occur in humans, where elevations in circulating acylcarnitines could be biomarkers of mitochondrial dysfunction [88], but only if measured prior to administration of NAC [88,90].

Enhanced mitochondrial peroxynitrite formation coupled with the inhibition of mitochondrial antioxidant systems due to protein nitration ultimately induces the mitochondrial permeability transition (MPT), whose early inhibition provided transient protection that was however not sustainable [91]. Ultimately, the persistent loss of mitochondrial membrane potential sustains the MPT [86,91,92], accompanied by mitochondrial fission mediated by canonical proteins involved in mitochondrial dynamics such as Drp1 [93]. The induction of the MPT allows the release of mitochondrial proteins such as endonuclease G (Endo G) and apoptosis-inducing factor (AIF) into the cytosol and their subsequent translocation to the nucleus [94,95]. Once within the nucleus, AIF induces chromatin condensation [96] and, in co-operation with Endo G, cleaves nuclear DNA, causing its fragmentation [96,97]. The partial protection against APAP-induced hepatocyte necrosis in AIF-deficient mice [98] highlights the role played by this mitochondrial protein in APAP pathophysiology. In addition to the MPT triggering the release of AIF and Endo G through matrix swelling and rupture of the outer membrane, mitochondrial Bax translocation and the formation of a Bax pore in the outer mitochondrial membrane can also induce the early release of these intermembrane proteins and cause DNA fragmentation [99]. In general, this type of mitochondria-dependent DNA fragmentation is the point-of-no-return in the intracellular signaling pathways to cell necrosis [100]. Thus, the mitochondria play a central role in APAP-induced hepatocyte necrosis, with the complex interplay of various signaling pathways converging on this organelle being continuously revealed by ongoing research in the field. However, the role of the mitochondria is not restricted to APAP-induced injury, as will be discussed in the following section.

## 5. Mitochondria in Liver Recovery and Regeneration

In addition to their central role in liver injury after APAP overdose, mitochondria also play critical roles in liver recovery after APAP-induced hepatocyte necrosis, with mitochondrial biogenesis being central to the process [101]. Acetaminophen-induced hepatocyte necrosis is characteristically centrilobular in nature, predominantly affecting hepatocytes surrounding the central vein, with mitochondrial spheroid formation and autophagy in cells beyond that, and mitochondrial biogenesis in cells farthest from the central vein [49]. Our earlier study demonstrated that mitochondrial biogenesis is induced in cells surrounding the area of necrosis beginning at 24 h after a 300 mg/kg overdose of APAP, accompanied by a substantial recovery in hepatic mtDNA levels [102]. The importance of the process for liver recovery after this moderate APAP overdose was illustrated by the enhanced recovery when mice were treated with the mitochondrial biogenesis inducer SRT1720 [102]. Mice deficient in fibroblast growth factor 21 (FGF21), which induces hepatocyte expression of PGC1α, the central regulator of mitochondrial biogenesis, also showed exacerbated liver injury after APAP overdose [103]. Induction of PGC-1α with inducers such as diphenyl diselenide was also able to enhance mitochondrial bioenergetics after APAP overdose [104]. Though mitochondrial biogenesis was not explicitly measured in these studies, the effects on PGC-1α will presumably influence mitochondrial biogenesis. Thus, induction of mitochondrial biogenesis in the discrete population of surviving hepatocytes surrounding areas of necrosis plays an essential role in liver recovery and regeneration after APAP overdose.

Another well-recognized factor in recovery and regeneration after APAP-induced liver injury is the innate immune response induced by the release of damage-associated molecular patterns (DAMPs) from necrotic hepatocytes, such as the high molecular weight group box 1 (HMGB1) protein [24,105]. These signals activate cytokine and chemokine formation in resident macrophages such as Kupffer cells, which then activate and recruit neutrophils, monocytes, and other leukocytes into the liver [105], which facilitate tissue repair and regeneration at moderate overdoses but can also aggravate the injury at severe overdoses [106]. Macrophages have high immune plasticity and their polarization is influenced by the microenvironment [107]. In the context of APAP, these monocyte-derived macrophages, though initially having a pro-inflammatory phenotype, mature after hepatic recruitment into a pro-regenerative phenotype with increased phagocytosis capacity and expression of anti-inflammatory genes [105]. It is recognized that parameters of cellular metabolism differ depending on macrophage phenotype, with pro-inflammatory macrophages mostly using glycolysis to meet their energetic needs, while anti-inflammatory macrophages rely on mitochondrial respiration, with changes in cellular metabolism influencing the cytokine secretion profile and expression of key inflammatory genes [107]. A recent study also found that myeloid-specific deletion of mitochondrial Complex I protein Ndufs4 (mKO) induced a proinflammatory metabolic profile in macrophages with a blunted transition to the reparative phase [108], reiterating the importance of macrophage mitochondrial function in the phenotype change. Our recent in vitro and in vivo experiments also demonstrated that Kupffer cells regulate CXCR2 expression and pro-regenerative gene expression in surviving hepatocytes around the areas of necrosis through the production of IL-10 to support the transition of these hepatocytes around the areas of necrosis to a proliferative state [109]. We further demonstrated that these recovered hepatocytes then promote macrophage apoptosis through CXCR4 signaling to resolve the inflammatory response and return to homeostasis [110]. Interestingly, it has also been demonstrated that neutrophils promote the development of reparative macrophages through ROS production to facilitate liver repair after an APAP overdose [111], and the role of mitochondria in controlling several facets of neutrophil physiology, including maturation and behavior, is now being recognized [112]. Thus, in addition to its role within hepatocytes through the induction of biogenesis, mitochondria could potentially have important roles within the infiltrating immune cells in controlling recovery and regeneration after an APAP overdose. Though information on these aspects is currently scarce, it is an important area for future investigation.

## 6. Conclusions

**Figure 1.**
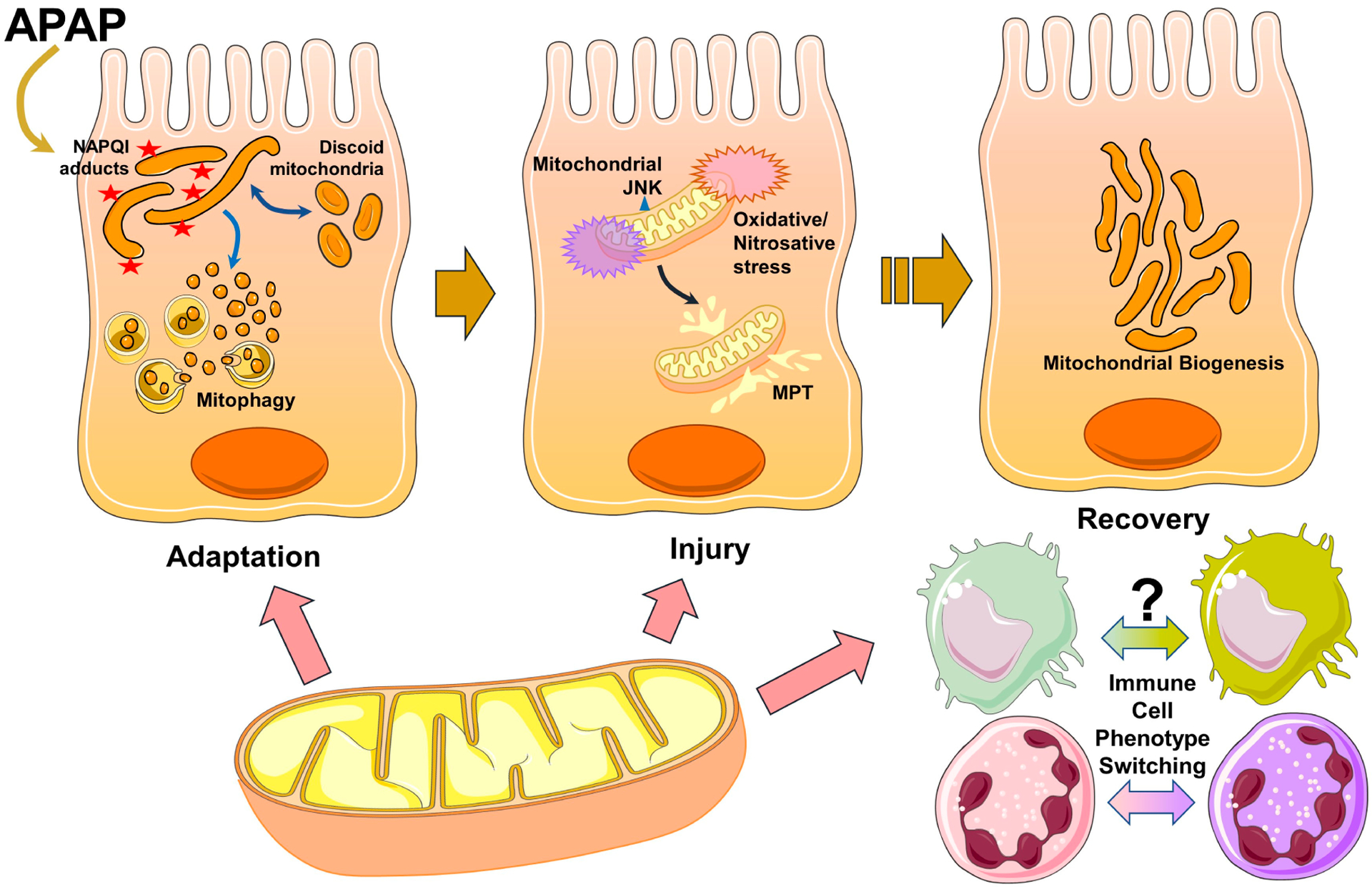
Mitochondria are involved in multiple phases of APAP pathophysiology. Formation of protein adducts on mitochondria due to generation of the reactive metabolite NAPQI from an acetaminophen (APAP) overdose initiates cellular stress. This induces early adaptive changes in mitochondrial morphology due to a decrease in membrane potential, which are reversible. Additional adaptive mechanisms include mitophagy, which allows the removal of dysfunctional mitochondria after their fragmentation. Persistence of adduct formation and activation of the MAP kinase JNK after APAP would then cause mitochondrial JNK translocation accompanied by oxidative and nitrosative stress in the organelle with induction of the mitochondrial permeability transition (MPT), which ultimately causes hepatocyte necrosis. In addition to these roles in adaptation and injury, mitochondria are also involved in liver recovery with the induction of mitochondrial biogenesis in surviving hepatocytes, facilitating liver regeneration. The organelle may also be involved in phenotype switching of infiltrating immune cells to the reparative phenotype to aid in liver recovery.

Taken together, it is now evident that mitochondria play central roles in both liver injury and recovery after an APAP overdose (Figure 1). While these aspects were recognized earlier, recent evidence revealed the nuanced response of the organelle to increased generation of the reactive metabolite NAPQI and indicates that significant attempts at adaptation to the insult are initiated. It is only when the persistent formation of mitochondrial protein adducts and JNK-mediated inhibition of electron transport overwhelm these adaptive mechanisms that the organelle undergoes MPT, triggers nuclear DNA fragmentation, and finally causes hepatocyte necrosis. From a therapeutic standpoint, enhancing these adaptive mechanisms could be an approach to delay the cascade of necrotic cell death and prevent progression to acute liver failure after APAP overdose. Additionally, further studies on the role of macrophage and neutrophil mitochondria in modulating the innate immune response would uncover additional avenues that could be targeted to facilitate recovery and regeneration after APAP overdose.
